# Exposure of Neonatal Rats to Parathion Elicits Sex-Selective Reprogramming of Metabolism and Alters the Response to a High-Fat Diet in Adulthood

**DOI:** 10.1289/ehp.11673

**Published:** 2008-06-23

**Authors:** T. Leon Lassiter, Ian T. Ryde, Emiko A. MacKillop, Kathleen K. Brown, Edward D. Levin, Frederic J. Seidler, Theodore A. Slotkin

**Affiliations:** 1 Department of Pharmacology and Cancer Biology, Duke University Medical Center, Durham, North Carolina, USA; 2 GlaxoSmithKline Inc., Research Triangle Park, North Carolina, USA; 3 Department of Psychiatry & Behavioral Sciences, Duke University Medical Center, Durham, North Carolina, USA

**Keywords:** developing rat, diabetes, glucose homeostasis, high-fat diet, insulin, lipid homeostasis, obesity, organophosphate insecticides, parathion

## Abstract

**Background:**

Developmental exposures to organophosphate pesticides are virtually ubiquitous. These agents are neurotoxicants, but recent evidence also points to lasting effects on metabolism.

**Objectives:**

We administered parathion to neonatal rats. In adulthood, we assessed the impact on weight gain, food consumption, and glucose and lipid homeostasis, as well as the interaction with the effects of a high-fat diet.

**Methods:**

Neonatal rats were given parathion on postnatal days 1–4 using doses (0.1 or 0.2 mg/kg/day) that straddle the threshold for barely detectable cholinesterase inhibition and the first signs of systemic toxicity. In adulthood, animals were either maintained on standard lab chow or switched to a high-fat diet for 7 weeks.

**Results:**

In male rats on a normal diet, the low-dose parathion exposure caused increased weight gain but also evoked signs of a prediabetic state, with elevated fasting serum glucose and impaired fat metabolism. The higher dose of parathion reversed the weight gain and caused further metabolic defects. Females showed greater sensitivity to metabolic disruption, with weight loss at either parathion dose, and greater imbalances in glucose and lipid metabolism. At 0.1 mg/kg/day parathion, females showed enhanced weight gain on the high-fat diet; This effect was reversed in the 0.2-mg/kg/day parathion group, and was accompanied by even greater deficits in glucose and fat metabolism.

**Conclusions:**

Neonatal low-dose parathion exposure disrupts glucose and fat homeostasis in a persistent and sex-selective manner. Early-life toxicant exposure to organophosphates or other environmental chemicals may play a role in the increased incidence of obesity and diabetes.

Obesity and consequent type II diabetes are rising at epidemic rates in the United States and many other countries around the world, burdening individual lives and the community with substantial morbidity and mortality, as well as attendant financial costs. Two of three U.S. adults are now classified as overweight (Ogden et al. 2006), contributing to a parallel increase in the incidence of type II diabetes (Onkamo et al. 1999; Zimmet et al. 2001). The potential role of early-life chemical exposures in obesity and diabetes has garnered far less interest in comparison to factors such as diet and lifestyle, genetics, race and ethnicity, and socioeconomic status (Frayling et al. 2007; Stein and Colditz 2004; Wang and Beydoun 2007), but there is increasing evidence that such exposures may have a significant impact. As just one example, it is clear that maternal smoking during pregnancy produces metabolic reprogramming that leads to subsequent risk of obesity in the offspring (Al Mamun et al. 2006; Chen et al. 2006; Gao et al. 2005; Power and Jefferis 2002; Toschke et al. 2003; von Kries et al. 2002). Recent attention has turned to the organophosphate pesticides, which represent 50% of all the insecticide use worldwide (Casida and Quistad 2004) and to which virtually all children are exposed (Morgan et al. 2005). There are epidemiologic links between pesticide exposure and diabetes (Morgan et al. 1980; Saldana et al. 2007), and the same subpopulations that have the highest rates of obesity—inner-city, low-socioeconomic-status, agricultural populations—are also those that have greater exposure to organophosphates and other pesticides (Curl et al. 2002; Eskenazi et al. 2007; Fenske et al. 2000; Perera et al. 2005; Petchuay et al. 2006; Rothlein et al. 2006; Whyatt et al. 2005). Superimposed on these relationships, children are exposed more than adults because of their higher food and water consumption per kilogram body weight and their higher surface-to-volume ratio (Garry 2004; Landrigan et al. 1999; National Research Council 1993; Physicians for Social Responsibility 1995; Whyatt et al. 2007).

Although most studies of organophosphates focus on their neurotoxicity, there is increasing evidence that these agents may also have a lasting impact on metabolic function. Animal studies confirm that organophosphate administration in adults can lead to enhanced weight gain (Meggs and Brewer 2007) and diabetes-like changes in hepatic energy metabolism (Abdollahi et al. 2004). These relationships may be even more important when exposure occurs during development. Fetal and neonatal rats exposed to chlorpyrifos showed excess weight gain and leptin dysregulation (Lassiter and Brimijoin 2008), in association with sensitization of hepatic cell signaling to gluconeogenic and lipolytic inputs (Meyer et al. 2004), culminating in a metabolic pattern characteristic of dyslipidemia and prediabetes (Slotkin et al. 2005). Neonatal treatment with different doses of diazinon, straddling the threshold for significant cholinesterase inhibition, produced a biphasic effect on metabolism and on the weight gain evoked by increased dietary fat intake (Roegge et al. 2008); after low-level neonatal exposures, diazinon enhanced the weight gain evoked by a high-fat diet in adulthood, whereas the interaction was blunted at higher exposure levels.

The present study focused on yet a third organophosphate, parathion, which is more potent toward systemic toxicity than chlorpyrifos or diazinon but that at low, subtoxic doses, also differs from them in many other aspects of developmental neurotoxicity (Slotkin et al. 2006a, 2006b, 2007a, 2007b, 2008b). Here, we evaluated the lasting effects after a brief period of neonatal parathion treatment straddling the dose threshold for the first overt signs of toxicity, as characterized by a loss of viability of approximately 5–10% (Slotkin et al. 2006a). Evaluations consisted of longitudinal measures of weight gain, with assessment of serum markers to characterize glucose and lipid metabolism. In addition, we evaluated the effects of switching the animals to a high-fat diet, looking specifically for whether early-life parathion exposure would change the weight gain and/or metabolic responses to increased fat intake.

## Materials and Methods

### Animal treatments and diet

All experiments were carried out humanely and with regard for alleviation of suffering, following protocols approved by the Duke University Institutional Animal Care and Use Committee and in accordance with all federal and state guidelines. Timed-pregnant Sprague–Dawley rats (Charles River, Raleigh, NC) were housed in breeding cages, with a 12 hr light–dark cycle and free access to water and food (LabDiet 5001; PMI Nutrition, St. Louis, MO). On the day after birth, all pups were randomized and redistributed to the dams with a litter size of 10 (5 males, 5 females) to maintain a standard nutritional status. Parathion (99.2% purity; Chem Service, West Chester, PA) was dissolved in dimethylsulfoxide to provide consistent absorption (Slotkin et al. 2006a, 2006b; Whitney et al. 1995) and was injected subcutaneously in a volume of 1 mL/kg once daily on postnatal days (PNDs) 1–4; control animals received equivalent injections of dimethylsulfoxide vehicle. Doses of 0.1 and 0.2 mg/kg/day were chosen because they straddle the threshold for barely detectable cholinesterase inhibition and the first signs of reduced weight gain or impaired viability (Slotkin et al. 2006a, 2006b). Brain cholinesterase inhibition 24 hr after the last dose of 0.1 mg/kg parathion is reduced 5–10%, well below the 70% threshold necessary for symptoms of cholinergic hyper-stimulation (Clegg and van Gemert 1999). Randomization of pup litter assignments within treatment groups was repeated at intervals of several days up until weaning, and dams were rotated among litters to distribute any maternal caretaking differences randomly across litters and treatment groups. Offspring were weaned on PND21.

For the body weight and serum measurements, there were 12 rats per treatment group for each sex, with no more than one male and one female derived from a given litter in each group. Individual body weights were recorded weekly; beginning immediately after weaning, animals were handled every few days to accustom them to removal from the cage and contact with investigators. On PND105 (postnatal week 15) and continuing through the end of the study (PND154; postnatal week 22), half the rats were switched to a high-fat diet (OpenSource D12330; Research Diets Inc., New Brunswick, NJ), providing 58% of total calories as fat; this starting point for dietary manipulation was chosen so that we could draw parallels with our earlier work on diazinon (Roegge et al. 2008). The remaining rats continued on the standard LabDiet 5001 diet, which provides 13.5% of total calories as fat. Food consumption was recorded twice weekly. During the 22nd postnatal week, rats were restrained in a polyfilm restraint cone (Harvard Apparatus, Holliston, MA), and blood was sampled by saphenous venipuncture into serum separator tubes (capiject T-MG; Terumo, Elkton, MD). Samples were allowed to clot for 30 min and were sedimented for collection of serum, which was then stored at −80°C. A second whole blood sample was hemolyzed in distilled water and sodium azide for glycated hemoglobin (HbA1c) measurement. Food was then removed from the cage overnight, and a third sample was obtained with rats in the fasted state (12–14 hr without food).

### Assays

Serum glucose, cholesterol, β-hydroxybutyrate, nonesterified fatty acids (NEFA), and triglycerides were determined with an Olympus Au640 Clinical Chemistry Analyzer (Olympus America Inc., Melville, NY). Serum insulin was measured using a radioimmunoassay kit (Millipore, Billerica, MA). The hemolyzed blood sample was assayed for the percentage of HbA1c using a ColumnMate analyzer (Helena Laboratories, Beaumont, TX).

### Data analysis

Data are presented as means and SEs. To establish the effects of parathion and its relationship to the high-fat diet and other variables, we first conducted a multivariate analysis of variance (ANOVA; data log-transformed because of heterogeneous variance), which encompassed all variables in a global test in order to avoid increased type 1 errors that might result from repeated testing of the data set. The factors were neonatal treatment, diet, sex, serum measurement, and feeding status (fed vs. fasted; repeated measure, because each animal was evaluated in both states). Data were then subdivided according to the interactions between the variables to permit lower-order ANOVAs; where permitted by the interaction terms, individual groups that differed from control were identified with Fisher’s protected least significant difference test. Significance was assumed at the level of *p* < 0.05. For interactions at *p* < 0.1, we also examined whether lower-order main effects were detectable after subdivision of the interactive variables (Snedecor and Cochran 1967). The criterion for interaction terms was not used to assign significance to the effects but rather to identify interactive variables requiring subdivision for lower-order tests of main effects of parathion, the variable of chief interest (Snedecor and Cochran 1967).

For convenience, some of the data are presented as percentages of control values or as differences between high-fat and normal diet, but the statistical evaluations were conducted on the original values.

## Results

### Body weight and food consumption

**Figure 1:**
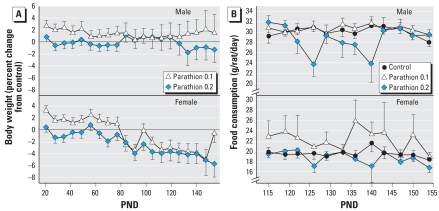
Effects of neonatal parathion exposure on body weights (*A*) and food consumption (*B*) in animals maintained on a normal diet. Body weight is shown as a percentage of age-matched controls. For reference, the control weights (grams) for each of the respective ages shown in the graph were males 50.5 ± 0.3, 95.8 ± 0.9, 154 ± 1, 217 ± 2, 276 ± 3, 334 ± 3, 379 ± 4, 412 ± 5, 441 ± 5, 463 ± 5, 493 ± 6, 507 ± 7, 530 ± 7, 540 ± 8, 549 ± 8, 565 ± 9, 574 ± 9, 584 ± 9, 593 ± 10, 601 ± 11; females, 48.6 ± 0.4, 89.1 ± 0.9, 133 ± 1, 170 ± 2, 193 ± 2, 209 ± 3, 232 ± 3, 245 ± 3, 258 ± 4, 268 ± 4, 282 ± 4, 284 ± 5, 293 ± 4, 296 ± 6, 299 ± 6, 306 ± 6, 311 ± 6, 317 ± 6, 314 ± 6, 314 ± 7. For body weights (*A*), ANOVA shows a significant main effect of parathion treatment (*p* < 0.0001) and an interaction of treatment × sex, *p* < 0.04. In males, the low-dose parathion group is significantly different from controls (*p* < 0.0002 for the main effect of parathion treatment). In females, the low dose parathion group shows a significant increase in body weight (*p* < 0.05 up to PND80), but starting in the 12th postnatal week, body weights are significantly subnormal for both parathion groups (*p* < 0.0001). For food consumption (*B*), ANOVA shows a main effect of parathion (*p* < 0.0001) and interactions of treatment × sex (*p* < 0.07) and treatment × age (*p* < 0.07). Males exposed to high-dose parathion show a significant treatment effect (*p* < 0.005) and interaction of treatment × age (*p* < 0.02). Females show a main effect of treatment (*p* < 0.0003) with higher food consumption in the low-dose group (*p* < 0.002).

Neonatal parathion exposure had no significant effect on body weight during the immediate treatment period (PNDs 1–4). However, it affected subsequent growth throughout juvenile, adolescent, and adult stages in a sex-selective manner (Figure 1A). In males, the low dose of parathion elicited a small but highly consistent and significant increase in body weight (*p* < 0.0002 for the main effect of parathion treatment), whereas at the higher dose, the weights were not significantly different from control values but were distinguishable from the increase seen in the low-dose group (*p* < 0.003). The weight differences in females showed a different profile, with significant deficits (*p* < 0.0009 for the main effect of parathion treatment); the effects generally intensified over time, so that although the low dose of parathion elicited an initial increase in body weight (up to PND80), starting in the 12th postnatal week, body weights became clearly subnormal across both parathion groups (Figure 1A). Because the effects on body weights could reflect underlying differences in food intake, we assessed consumption in postnatal weeks 15–22 (Figure 1B). In males, the low dose of parathion did not affect food consumption but animals exposed to the high dose showed reduced intake (treatment, *p* < 0.005; treatment × age, *p* < 0.02), primarily during postnatal weeks 18–20 (PNDs 126–140; treatment, *p* < 0.0002). Females showed a different pattern (treatment, *p* < 0.0003) with higher food consumption in the 0.1-mg/kg parathion group (*p* < 0.002 for the main effect of parathion treatment) and normal food consumption in the high-dose group.

**Figure 2:**
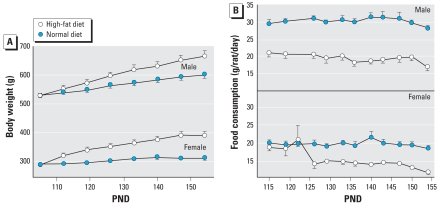
Effects of switching to a high-fat diet on PND105. (*A*) Body weight. (*B*) Food consumption. For body weight, ANOVA indicates a main effect of diet (*p* < 0.0001) and interactions of diet × sex (*p* < 0.0001) and diet × age (*p* < 0.03), significant for both sexes individually (*p* < 0.0001 for each). For food consumption (*B*), ANOVA indicates a main effect of diet (*p* < 0.0001) and interactions of diet × sex (*p* < 0.0005) and diet × age (*p* < 0.0001). For both sexes, there were main effects of diet (*p* < 0.0001) and interactions of diet × age (*p* < 0.0001 for males, *p* < 0.003 for females).

In control rats, introduction of a high-fat diet starting on PND105 led to increased weight gain (Figure 2A) and decreased food consumption (Figure 2B). In males, high-fat intake produced a progressive increase in weight, which by postnatal week 22 reached about 10% above the values for age-matched controls consuming a normal diet (*p* < 0.0001; Figure 2A). In females, the effect showed a quicker onset and was much larger, with almost a 30% increase in body weight by the end of the study (*p* < 0.0001). In compensation for the increased dietary caloric content, food consumption was reduced in both sexes (Figure 2B). In males, the reduced food intake began immediately upon introduction of the high-fat diet and intensified somewhat with time (Figure 2B). However, females on the high-fat diet maintained their food intake initially and showed a decreased consumption only after a lag of several weeks (Figure 2B), likely contributing to their more rapid onset of extra weight gain.

**Figure 3:**
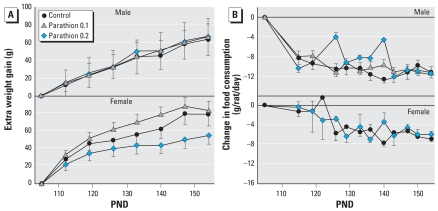
Interaction of neonatal parathion exposure with a high-fat diet introduced on PND105, presented as the difference between the high-fat diet and the normal diet. (*A*) Extra weight gain. (*B*) Change in food consumption. For weight gain (*A*), ANOVA indicates interactions of treatment × diet (*p* < 0.03) and treatment × diet × sex (*p* < 0.02); the parathion effect was significant in females (treatment, *p* < 0.0002), with the low doses enhancing the weight gain caused by the high-fat diet (treatment × diet, *p* < 0.09 vs. control; *p* < 0.0001 vs. 0.2 mg/kg/day parathion), whereas exposure to 0.2 mg significantly reduced the dietary effect (treatment × diet, *p* < 0.03 vs. control, *p* < 0.0001 vs. 0.1 mg/kg/day parathion). For food consumption (*B*), significant effects were confined to males exposed to 0.2 mg/kg/day parathion (treatment × diet, *p* < 0.009).

Early-life exposure to parathion affected the weight gain imposed by the introduction of a high-fat diet in adulthood (Figure 3A). In males, parathion did not alter the additional weight gain from the high-fat diet; by the end of the study, all neonatal treatment groups gained 60 g more than animals on the normal diet. In females, however, there was a biphasic effect with increasing parathion dose (treatment, *p* < 0.0002). Exposure to 0.1 mg/kg/day of parathion tended to enhance the weight gain caused by the high-fat diet, whereas exposure to 0.2 mg significantly reduced the dietary effect (Figure 3A). Food consumption also showed major sex differences in the effects of neonatal parathion exposure on the response to a high-fat diet (Figure 3B). Males exposed to 0.2 mg/kg/day parathion showed less of a decrease in food consumption on the high-fat diet than did controls, with the effect exhibited primarily during weeks 18–20, midway through the period of dietary manipulation. In contrast, females exposed to 0.2 mg/kg/day parathion showed exactly the same pattern of decreased food intake as controls when placed on a high-fat diet. We could not obtain reliable food consumption in the low-dose females given the high-fat diet because this group uniquely wasted large amounts of food, as evidenced by accumulation of debris under the cage. It is not clear if this is a behavioral difference or simply an anomaly, but we did observe this behavior in all but one cage in this group, and the effect was consistent across all the age points.

### Glucose homeostasis

**Figure 4:**
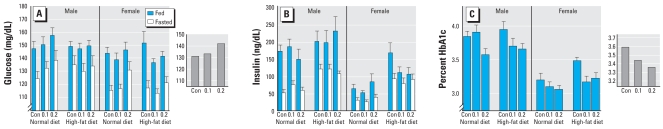
Serum measures related to glucose metabolism in the fed and fasted states in animals exposed to neonatal parathion, followed by normal or high-fat diets in adulthood. Con, control. (*A*) Glucose. (*B*) Insulin. (*C*) Glycated hemoglobin (HbA1c). For glucose (*A*), ANOVA indicated a significant main effect of parathion for animals on a normal diet (*p* < 0.05). Fasting, by itself, decreased glucose levels (*p* < 0.0001) without altering the treatment effect of parathion (no interaction of treatment × feeding status). The inset shows the values for parathion effects on a normal diet, collapsed across sex and feeding status, and calculated as the geometric mean of all values. For insulin levels (*B*), parathion had no significant effect, but levels were reduced by fasting (*p* < 0.0001) and increased by diet (*p* < 0.0001) in a sex-selective fashion (diet × sex, *p* < 0.03). For HbA1c (*C*) ANOVA indicates a significant main effect of parathion (*p* < 0.002) that was individually significant for both 0.1 mg/kg/day (*p* < 0.02) and 0.2 mg/kg/day groups (*p* < 0.0005). There was also a main effect of sex (*p* < 0.0001), but effects were significant for both males (*p* < 0.03) and females (*p* < 0.03). The inset shows the values for parathion effects collapsed across sex and diet, and calculated as the geometric mean of all values.

On a normal diet, neonatal parathion exposure had an effect on circulating glucose levels, with the higher dose eliciting a small, but statistically significant increase (main effect, *p* < 0.02) that was not sex-selective (no treatment × sex interaction; Figure 4A). As expected, fasting reduced glucose levels significantly, but this did not eliminate the increase caused by neonatal parathion exposure (no interaction of treatment × feeding status). To illustrate the main effect of parathion, we collapsed the values across the noninteractive variables (sex, feeding status), calculating the geometric mean of all values in each treatment group (Figure 4A inset). Introduction of a high-fat diet did not by itself cause a significant change in glucose levels, but it did lessen the hyperglycemic effect of neonatal parathion exposure to the point where the parathion effect was no longer statistically significant.

Despite the hyperglycemia caused by neonatal parathion exposure, we did not find any significant effects of parathion alone on insulin levels in animals given a normal diet (Figure 4B); as expected, insulin levels fell in the fasting state. Introduction of a high-fat diet evoked a significant increase in insulin levels (*p* < 0.0001 for the main effect of diet), with a relatively larger increase in females than in males (Figure 4B). None of these relationships showed any statistically significant parathion-related changes.

Since glycation of hemoglobin takes place over a long time, we assessed HbA1c only in the fed state. Parathion evoked a significant reduction in HbA1c (Figure 4C). There was also a strong sex difference, with males having higher values than females; although there was a marginal interaction of treatment × sex (*p* < 0.08), the effects were seen in both males (*p* < 0.03) and females (*p* < 0.03), and the same anomaly was seen after introduction of a high-fat diet. To illustrate the main effect of parathion, we collapsed the values across the noninteractive variables (sex, diet), calculating the geometric mean of all values in each treatment group (Figure 4C inset).

### Lipid homeostasis

**Figure 5:**
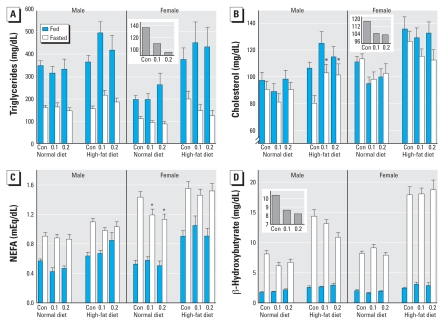
Serum measures related to lipid metabolism, taken in the fed and fasted states from animals exposed to neonatal parathion, and given normal or high-fat diets in adulthood. Con, control. (*A*) Triglycerides. (*B*) Cholesterol. (*C*) Nonesterified fatty acids. (*D*) β-Hydroxybutyrate. For triglycerides (*A*), ANOVA shows main effects of diet (*p* < 0.0001), sex (*p* < 0.0001), and feeding status (*p* < 0.0001), and an interaction for parathion × sex × feeding status (*p* < 0.08). Lower-order ANOVAs indicate a significant treatment × sex interaction (*p* < 0.04) in the fasted state, with a significant effect limited to females (*p* < 0.05 for the main effect of parathion; *p* < 0.02 for the high-dose parathion group). The inset shows the values for parathion effects in fasted females collapsed across diet, and calculated as the geometric mean of all values. For cholesterol (*B*), ANOVA indicates main effects of diet (*p* < 0.0001), sex (*p* < 0.0001) and feeding status (*p* < 0.0001) and interactions of parathion treatment with all three variables: treatment × diet (*p* < 0.08), treatment × sex (*p* < 0.1) and treatment × sex × feeding status (*p* < 0.1). Lower-order ANOVAs indicate a significant effect of parathion in fasted males on the high-fat diet (asterisks). For females, parathion had a significant effect on fasting cholesterol (*p* < 0.05) without dependence on diet; the inset shows the values for parathion effects in fasted females collapsed across diet, and calculated as the geometric mean of all values. For NEFA (*C*), ANOVA indicates a main effect of feeding status (*p* < 0.0001), an interaction of feeding status × diet (*p* < 0.0002) and significant effects of parathion treatment on a normal diet (main effect, *p* < 0.04), limited to females (*p* < 0.04, asterisks). For β-hydroxybutyrate (*D*), ANOVA shows main effects of feeding status (*p* < 0.0001), sex (*p* < 0.0003), and diet (*p* < 0.0001), with parathion interactions with all three variables (*p* < 0.06 for treatment × feeding status, *p* < 0.08 for treatment × sex × feeding status, *p* < 0.09 for treatment × sex × diet × feeding status). Lower-order ANOVAs indicated significant effects in the fasted state, restricted to males (main effect of parathion, *p* < 0.007); the inset shows the values for parathion effects in fasted males collapsed across diet, and calculated as the geometric mean of all values.

Switching to a high-fat diet produced the expected increases in triglycerides regardless of sex, feeding status, or neonatal parathion exposure (Figure 5A); superimposed on the dietary effect, males had higher values than females, and fasting reduced the levels. Neonatal parathion exposure altered the triglyceride pattern in a sex-selective manner that also depended on whether animals were fed or fasted (Figure 5A). Accordingly, since feeding status itself had a significant effect on triglycerides, we evaluated effects separated into fed and fasted values and then identified a significant treatment × sex interaction (*p* < 0.04) for the fasted state; this reflected a significant decrease in females (Figure 5A). To illustrate the main effect of parathion in fasted females, we collapsed the values across diet, calculating the geometric mean of all values in each treatment group (Figure 5A inset).

Cholesterol (Figure 5B) likewise showed large increases evoked by the high-fat diet, lower values in males than females, and lower values in the fasted state. Neonatal parathion treatment showed interactions with all the other variables. Given the large effect of feeding status, we again separated the fed from the fasted values and identified treatment effects largely confined to the fasted groups and showing sex differences (treatment × sex, *p* < 0.04). In males on a normal diet, neonatal parathion had little or no effect on fasting serum cholesterol values; however, differences emerged when animals were switched to a high-fat diet, with significantly higher cholesterol in the parathion-exposed males (*p* < 0.05). On the other hand, females exposed to neonatal parathion showed a reduction in fasting cholesterol (*p* < 0.05) without clear distinction between normal and high-fat diets (no treatment × diet interaction). To illustrate the main effect of parathion in fasted females, we collapsed the values across diet, calculating the geometric mean of all values in each treatment group (Figure 5A inset).

By itself, overnight fasting produced substantial net increases in serum NEFA, and feeding status interacted with diet (*p* < 0.0002). Accordingly, we again separated fasting from fed values for evaluation of parathion effects (Figure 5C). In the fed state, neonatal parathion exposure had little or no discernible effect. In the fasted state, animals on a normal diet showed a reduction in NEFA caused by parathion, with significant effects limited to females. Upon introduction of the high-fat diet, these differences were no longer evident.

Serum β-hydroxybutyrate showed massive increases with overnight fasting (Figure 5D), in keeping with a switch in energy utilization from glucose to fat. Values were higher in females and were enhanced by a high-fat diet. The effects of neonatal parathion treatment interacted with each of the other variables. Separating the values for fed and fasted animals, we again observed parathion effects that were restricted to the fasted state. Males exposed to parathion showed lower β-hydroxybutyrate values than fasted controls (*p* < 0.007), whereas females did not. The effect did not depend on whether the animals consumed a normal or high-fat diet (no interaction of treatment × diet). To illustrate the main effect of parathion in fasted males, we collapsed the values across diet, calculating the geometric mean of all values in each treatment group (Figure 5D inset).

## Discussion

In our earlier work with chlorpyrifos, we found that neonatal exposure altered the programming of metabolic function, enhancing hepatic responses to inputs mediating gluconeogenesis and lipolysis (Meyer et al. 2004), with glucose homeostasis maintained only through increased circulating insulin levels and attendant hyperlipidemia, essentially producing a prediabetic state (Slotkin et al. 2005). With more prolonged developmental exposures, low doses of chlorpyrifos enhanced adiposity and weight gain while dysregulating leptin, whereas the effect was lost at higher doses that exceeded the threshold for growth impairment and other signs of systemic toxicity (Lassiter and Brimijoin 2008). In all these cases, males showed more prominent effects than females. Here, in a parallel examination of the effects of neonatal parathion exposure, we similarly found a biphasic relationship in animals maintained on a normal diet. At the lower dose, below the threshold for signs of systemic toxicity, the parathion-exposed males displayed essentially an anabolic response, increasing their weight gain without a corresponding change in food consumption, and with a reduced mobilization of fat upon fasting, as evidenced by smaller increase in β-hydroxybutyrate compared with the response in controls. When the neonatal parathion dose was increased to 0.2 mg/kg/day, the subsequent anabolic response was lost, implying negative effects on energy metabolism at these higher exposures. This raises the critical question of what mechanisms mediate the reversal of the anabolic actions. One important clue is provided by the parallel findings of increased plasma glucose but a reduction in HbA1c. Ordinarily, HbA1c increases in response to chronic or repeated episodes of hyperglycemia, but the fact that we saw a decrease implies that there is a deficiency in the uptake or utilization of glucose by the cell. This recapitulates one of the functional end points of diabetes, “starving amid plenty,” albeit by a different underlying mechanism. Notably, the outcome after parathion exposure is distinct from that seen with chlorpyrifos, where plasma glucose is kept in check by insulin hypersecretion (Slotkin et al. 2005); the two organophosphates produce diabetes-like effects but with different specific characteristics centered around circulating insulin, glucose, and lipids.

In contrast to the anabolic effects of low-level parathion exposure in males, in females, we found catabolic effects, characterized by weight deficits, at both doses. This suggests that females are more sensitive to the negative metabolic and growth effects of parathion than males. Indeed, in the low-dose group, the body weight deficits were superimposed on increased food consumption, so that they had a growth deficiency despite higher caloric intake. Females displayed the same defects in glucose metabolism as males, with elevated fasting glucose levels, no compensatory increase in insulin, and reduced HbA1c pointing to deficient cellular uptake/utilization of glucose. Accordingly, females must have further metabolic defects to explain their greater sensitivity than males, and indeed, these became evident when we examined serum markers of lipid metabolism. Parathion-exposed females displayed decreased levels of triglycerides, cholesterol, and NEFA, implying an ongoing reduction in fat utilization, effects that were not seen in males. Further, with fasting, females exposed to parathion did not show the reduction in β-hydroxybutyrate production that was seen in males, again indicating an sex difference in the effects on the ketogenic response to food deprivation.

These findings all point to more extensive alterations in lipid metabolism caused by neonatal parathion exposure in females, associated with increased susceptibility to metabolic reprogramming that resulted in a net catabolic response, as opposed to the biphasic response seen in males. If this hypothesis is correct, we would expect to see even greater divergence between the sexes on a high-fat diet. The high-fat diet raised serum insulin, triglycerides, cholesterol, NEFA, and β-hydroxybutyrate, all in keeping with an increase in calories derived from fat. However, even in controls, there were significant sex differences in the response to increased fat intake, with females gaining a relatively greater proportion of body weight. Regarding parathion effects, in males, the switch to a high-fat diet revealed further metabolic defects beyond those seen with a normal diet. Although parathion did not affect the extra weight gain from high fat, males exposed to high-dose parathion showed a smaller reduction in food intake, so they were consuming more calories in order to grow at the same rate as the other groups; this implies a worsening of the catabolic effects compared to those on a normal diet. The serum markers point to defects in lipid metabolism as the underlying cause of the catabolic effects. Whereas only one parameter of lipid metabolism was affected by parathion in males on a normal diet (β-hydroxybutyrate response to fasting), switching to the high-fat diet revealed additional abnormalities, namely an increase in fasting cholesterol and a greater deficit in the β-hydroxybutyrate response; the latter potentially connotes an impaired ability to turn on ketogenesis when required by food deprivation. Finally, parathion-exposed males switched to the high-fat diet still showed a significant reduction in HbA1c (despite normal circulating glucose levels), reinforcing that the underlying defect in glucose uptake/utilization was still present.

Females showed even greater interactions between neonatal parathion exposure and the subsequent introduction of a high-fat diet. Low-dose parathion increased the weight gain caused by elevated fat intake, whereas high-dose parathion reduced it; the latter effect was seen even though the animals consumed the same amount of food (calories), implying further induction of a catabolic state. As in the males, the high-fat diet eliminated the increase in serum glucose but did not alter the underlying defect in glucose uptake/utilization, as evidenced by persistence of a decrease in HbA1c. In females, the increased fat intake reversed the deficiencies in NEFA but worsened the effects on triglycerides and cholesterol; the metabolic mechanisms underlying these interactions remain to be elucidated, but they do imply that the parathion-induced alterations of lipid metabolism contribute to the altered weight gain caused by the high-fat diet.

## Conclusions

Our results point to lasting metabolic dysregulation as a consequence of neonatal parathion exposure, characterized in males by a net anabolic response at low exposures and a catabolic response at higher exposures, and in females by a greater sensitivity to catabolic effects. Furthermore, there are specific interactions with dietary fat intake that can worsen the outcome. The anabolic effects raise the potential for increased weight gain and potential exacerbation by a high-fat diet; further, the metabolic alterations are consistent with a pre-diabetic state or with end points that are functionally similar to diabetes (elevated glucose, lipids, reduced ability of the cell to take up or utilize glucose). Parathion thus shares some, but not all the characteristics of the metabolic effects of other organophosphates such as chlorpyrifos (Lassiter and Brimijoin 2008; Slotkin et al. 2005) or diazinon (Roegge et al. 2008), reinforcing the fact that although these agents are all in the same class of pesticides and all inhibit cholinesterase, they also display individual effects that are unrelated to that shared property. Thus, organophosphates are not all the same in their metabolic effects, echoing findings for their individual actions as developmental neurotoxicants (Jameson et al. 2007; Levin et al. 2001; Pope 1999; Roegge et al. 2008; Slotkin et al. 2006a, 2006b, 2007a, 2008a; Slotkin and Seidler 2007; Timofeeva et al. 2008). Although we have not yet pursued the specific cellular mechanisms underlying these differences, one likely possibility is divergence in the effects on cell signaling cascades mediating gluconeogenesis and lipolysis, notably the adenylyl cyclase/cyclic AMP pathway (Meyer et al. 2004), and studies are under way to evaluate this possibility.

Our most important findings center on the tendency to categorize environmental toxicants by allocating them to preconceived classes. Organophosphates are usually thought of as developmental neurotoxicants, but they obviously have other important targets that contribute to morbidity, including metabolic effects that can have a potential impact on obesity and diabetes. It is increasingly evident that adverse events in fetal or neonatal life, including chemical exposures like those studied here, can lead to misprogramming of metabolism, appetite, and endocrine status contributing ultimately to morbidities such as obesity and diabetes (Breier et al. 2001). Clearly, we need to focus further research on the specific contributions of environmental chemical exposures that might be contributing to the epidemic of these and other metabolic disorders.
